# Comparison of Short- and Long-Term Effectiveness between Anti-TNF and Ustekinumab after Vedolizumab Failure as First-Line Therapy in Crohn’s Disease: A Multi-Center Retrospective Cohort Study

**DOI:** 10.3390/jcm12072503

**Published:** 2023-03-26

**Authors:** Ahmad Albshesh, Lian Bannon, Tali Sharar Fischler, Marie Truyens, Stephan R. Vavricka, Katja Tepes, Daniela Pugliese, Edoardo V. Savarino, Eran Zittan, David Drobne, Xavier Roblin, Ariella Bar-Gil Shitrit, Alessandro Armuzzi, Triana Lobaton, Nitsan Maharshak, Henit Yanai, Shomron Ben-Horin, Uri Kopylov

**Affiliations:** 1 Sheba Medical Center, Department of Gastroenterology, Tel Hashomer, Ramat Gan 5262100, Israel; 2 Sackler School of Medicine, Tel-Aviv University, Tel-Aviv 52621, Israel; 3 Tel Aviv Medical Center, Department of Gastroenterology and Liver Diseases, Tel-Aviv 6423906, Israel; 4 Rabin Medical Center, Division of Gastroenterology, Department of Gastroenterology, Petah Tikva 49100, Israel; 5 IBD Unit, Department of Gastroenterology, Ghent University Hospital, 9000 Ghent, Belgium; 6 Division of Gastroenterology and Hepatology, University Hospital, CH-8048 Zurich, Switzerland; 7 General Hospital Celje, 3000 Celje, Slovenia; 8 IBD Unit, Fondazione Policlinico Universitario A. Gemelli IRCCS, Università Cattolica del Sacro Cuore, 00168 Rome, Italy; 9 Division of Gastroenterology, Department of Surgery, Oncology and Gastroenterology, University of Padua, 35128 Padua, Italy; 10 Abraham and Sonia Rochlin IBD Unit, Department of Gastroenterology, Emek Medical Center, Afula 1834111, Israel; 11 Rappaport Faculty of Medicine Technion, Israel Institute of Technology, Haifa 3200003, Israel; 12 Department of Gastroenterology, University Medical Centre Ljubljana, 1231 Ljubljana, Slovenia; 13 Medical Faculty, University of Ljubljana, 1231 Ljubljana, Slovenia; 14 Service de Gastrologie-Entérologie-Hépatologie, CHU de Saint-Etienne, 42270 Saint-Etienne, France; 15 Shaare Zedek Medical Center, Digestive Diseases Institute, Jerusalem 9103102, Israel; 16 Faculty of Medicine, Hebrew University of Jerusalem, Jerusalem 9372212, Israel; 17 IBD Center, IRCCS Humanitas Research Hospital, Rozzano, 35128 Milan, Italy; 18 Department of Biomedical Sciences, Humanitas University, Pieve Emanuele, 20090 Milan, Italy

**Keywords:** Crohn’s disease, drug positioning, treatment response, treatment failure, ustekinumab, vedolizumab, anti-TNF

## Abstract

Background: The effectiveness of anti-TNF or ustekinumab (UST) as a second-line biologic after vedolizumab (VDZ) failure has not yet been described. Aims and Methods: In this retrospective multicenter cohort study, We aim to investigate the effectiveness of anti-TNF and UST as second-line therapy in patients with Crohn’s disease (CD) who failed VDZ as a first-line treatment. The primary outcome was clinical response at week 16–22. Secondary outcomes included the rates of clinical remission, steroid-free clinical remission, CRP normalization, and adverse events. Results: Fifty-nine patients who failed on VDZ as a first-line treatment for CD were included; 52.8% patients received anti-TNF and 47.2% UST as a second-line therapy. In initial period (Week 16–22), the clinical response and remission rate was similar between both groups: 61.2% vs. 68%, *p* = 0.8 and 48.3% vs. 56%, *p* = 0.8 on anti-TNF and UST therapy, respectively. Furthermore, in the maintenance period the rate was similar: 75% vs. 82.3%, *p* = 0.8 and 62.5% vs. 70.5%, *p* = 0.8, respectively. Of the patients, 12 out of the 59 stopped the therapy, without a significant difference between the two groups (*p* = 0.6). Conclusion: Second-line biological therapy after VDZ failure therapy was effective in >60% of the patients with CD. No differences in effectiveness were detected between the use of anti-TNF and UST as a second line.

## 1. Introduction

Biological therapies have revolutionized the management of Crohn’s disease (CD). Monoclonal antibodies against tumor necrosis factor alpha (anti-TNF) have been the corner stone of CD therapy since start the century [1,2,3,4]. More recently two additional biologicals, with different mechanism of action, have been added to the CD armamentarium: vedolizumab (VDZ) (α4β7 integrin inhibitor) [5,6,7,8,9,10] and ustekinumab (UST) (anti-interleukin-12/23) [2,6,7,9,11,12,13,14,15,16]. Despite the good efficacy, loss of response remains a serious concern across all therapeutic agents; the annual risk of loss of response to anti-TNF approaches 20–37% [17,18]. A similar rate of loss of response was described also in VDZ and UST [15,16,17,18,19,20]. In the last few years VDZ has been used as first-line therapy in CD with similar effectiveness as anti-TNF and very good safety profile [9,21,22,23].

With the advent of more agents, there is a dire need to develop not only positioning but also sequencing strategies. Nonetheless, there is limited guidance on optimal choice of agents as second-line therapies because of the absence of head-to-head trials after VDZ failure [3,24,25,26]. Accordingly, the aim of this study was to assess comparative effectiveness in terms of the induction of clinical response and remission with respect to anti-TNF versus UST in CD patients who showed failure of VDZ therapy. In addition, we attempted to identify predictors for the induction and maintenance of remission.

## 2. Materials and Methods

This was a multicenter retrospective cohort study. We included adult patients with an established CD diagnosis who received VDZ as a first-line therapy and switched to a second-line therapy that was either anti-TNF or UST. Clinical, endoscopic and laboratory data were extracted from the patient’s files and electronic records.

Patients eligible for inclusion in the study had to satisfy the following criteria: adult patients (≥18 years) with a confirmed CD who received VDZ as a first-line therapy and were switched to either anti-TNF or UST for a second-line therapy. All patients must have active disease and had follow-up for a minimum of 16 weeks after starting anti-TNF or UST therapy, patients who failed the therapy prior to week 16 were also included. Patients with under 16 weeks of follow-up post induction, with ostomy or a change in diagnosis to ulcerative colitis or inflammatory bowel disease unclassified (IBD-U) were excluded.

Demographic and clinical information that was collected included: age, gender, smoking status, type of anti-TNF agent, date of diagnosis, disease duration, disease location and Behavioral (Montreal classification), presence of perianal disease, duration of treatment with VDZ, concomitant immunomodulator at initiation of second-line therapy, corticosteroid use, C-reactive protein (CRP), fecal calprotectin (FCP), Harvey–Bradshaw Index (HBI), requirement for subsequent hospitalization, surgery and adverse events.

The study was approved by the Sheba Medical Center ethics committee. The patients’ consent was waived.

### 2.1. Outcome Definitions

The primary outcome was defined as a clinical response (defined by a reduction of HBI ≥ 3) [27] at week 16. Clinical response was assessed at the following time-points: initial response (week 16–22) and maintenance response (week 52).

Main secondary outcomes included the following in the two time points after induction: clinical remission (HBI ≤ 4) [27]; steroid-free clinical remission; drug discontinuation; CRP normalization ((CRP serum concentration levels less than normal range as per the cut-off used in the corresponding institutions) in patients with elevated baseline CRP; FCP normalization (<250 µg/mg) in patients with elevated baseline FCP.

Clinical and demographic characteristics were compared between the patients who received anti-TNF and UST, in order to identify potential predictive factors for clinical response. An adverse event is defined as any adverse reaction that occurs after initiating treatment.

### 2.2. Statistical Methods

The primary and secondary end points were calculated and compared for both the anti-TNF and UST patient groups. All variables were reported as mean ± standard deviation (SD) or proportions. Between-group comparisons were performed using unpaired t tests, Chi-square, Fisher’s exact or Wilcoxon rank testing, as appropriate. A survival analysis curve with COX regression was constructed for analysis of time to treatment discontinuation. The model was adjusted for age at diagnosis, disease behavior, presence of perianal disease, and steroid use at baseline. *P* values of less than 0.05 were considered statistically significant. Statistical analysis was performed using IBM SPSS Statistics software version 22. The data-extraction sheets contained only anonymized data.

## 3. Results

### 3.1. Baseline Characteristics

**Figure 1:**
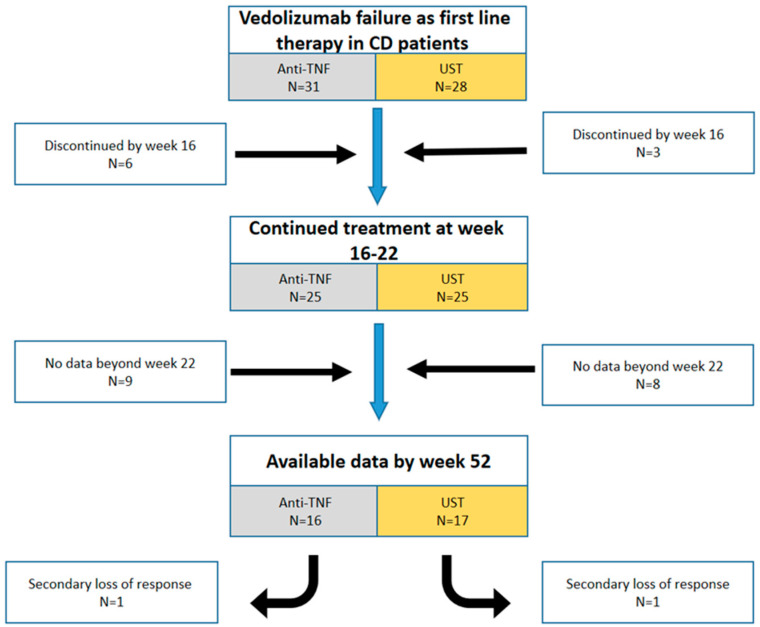
Chart of the patients’ flow during the study period. Abbreviations: CD, Crohn’s disease; anti-TNF, anti-Tumor Necrosis Factor; UST, ustekinumab.

Fifty-nine patients from eleven centers in six different countries (six Europe, five Israel) were included in the study. The clinical and demographic characteristics of the patients are detailed in Table 1. All patients received VDZ as a first-line biological therapy and experienced therapy failure. The median time of VDZ treatment was 13.5 months (interquartile range (IQR) 5–18). The main reason for the discontinuation of VDZ was a lack of response (clinically, endoscopic and biomarker non- response (47.6%, 32.4% and 6.8%, respectively)). The rest discontinued the treatment because of: adverse event (3.3%), active extraintestinal manifestation (3.3%), surgery (3.3%) and active perianal disease (3.3%). Active disease was the indication for the second-line therapy initiation. A total of 31 out of the 59 patients were switched to anti-TNF (61.3% infliximab, 35.5% adalimumab, 3.2% certolizumab), and the remaining 28 patients were switched to UST (52.8% vs. 47.2%, respectively (*p* = 0.7)). There were no significant differences between the two groups as detailed in Table 1. Figure 1 describes the patient flow during the study.

### 3.2. Treatment Outcomes

#### 3.2.1. Initial Response

**Figure 2:**
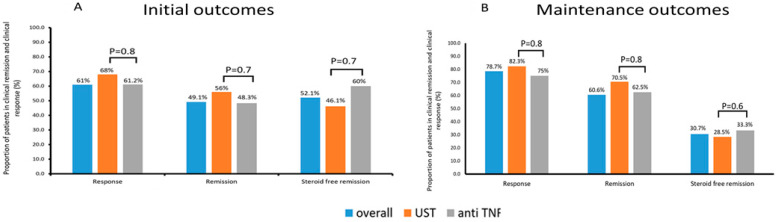
Outcomes in the initial (**A**) and maintenance period (**B**). The proportion of patients achieving clinical remission (HBI ≤ 4), clinical response (delta HBI ≥ 3) and steroid-free remission during the initial period (week 16–22) (**A**), and the proportion of patients achieving clinical remission, clinical response and steroid free remission during the maintenance period (week 52) (**B**); HBI: Harvey–Bradshaw index; UST: ustekinumab; anti-TNF: anti-tumor necrosis factor.

An initial response (week 16–22) was achieved in 61% of the patients (19/31 (61.2%) and 17/25 (68%) on anti-TNF and UST therapy, respectively (*p* = 0.8)). Rates of clinical remission were similar between both groups (15/31 (48.3%) and 14/25 (56%), (*p* = 0.7). Systemic corticosteroids were discontinued in 16/23 (69.5%) patients who were on corticosteroids at the treatment onset. Twelve out of 23 (52.1%) patient achieved corticosteroid-free remission (6/10 (60%) and 6/13 (46.1%), (*p* = 0.7) (Figure 2).

#### 3.2.2. Maintenance of Response

Follow up results in the maintenance period (median 53 weeks [IQR 48–58]) were available for 33/59 patients (16 (48.5%) and 17 (51.5%), respectively). A further 26 patients were excluded from the analysis of this time period (for 17 patients no data were available and 9 discontinued the therapy by week 16 (Figure 1).

The overall response rate was 78.7% (12/16 (75%) and 14/17 (82.3%) on anti-TNF and UST therapy, respectively (*p* = 0.8)). Clinical remission was achieved by 22/33(60.6%) patients (10/16 (62.5%) and 12/17 (70.5%), (*p* = 0.8), respectively). Of the 23 patients who were on corticosteroids at the treatment onset, 13 had available follow up results. The corticosteroid discontinuation rate was 58.3% in patients who were on corticosteroids at treatment onset. Corticosteroid-free remission was achieved by 4/13 (30.7%) patients (2/6 (33.3%) and 2/7 (28.5%), (*p* = 0.9)) (Figure 2).

#### 3.2.3. Changes in CRP and FCP

**Figure 3:**
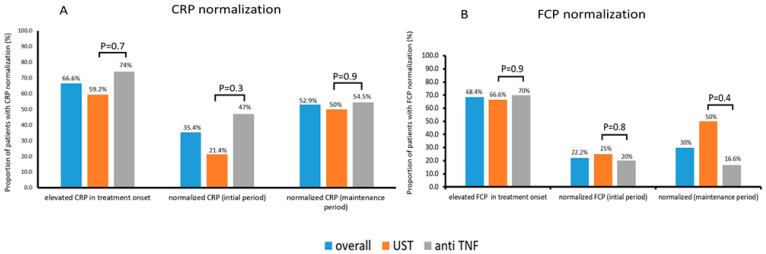
Biomarker normalization. The proportion of patients with CRP normalization (**A**) (CRP serum concentration levels less than normal range as per cut-off used in the corresponding institutions) and FCP normalization (**B**) (FCP < 250 µg/g); CRP, C-reactive protein; FCP, Fecal Calprotectin; UST, ustekinumab; anti-TNF, anti-tumor necrosis factor.

CRP levels were available in 54 out of the 59 patients and were elevated in 36 (66.6%) of them at treatment onset (20/27 (74%) and 16/27 (59.2%), on anti-TNF and UST therapy, respectively (*p* = 0.7)). CRP normalized in 11/31 (35.4%) patients (8/17 (47%) and 3/14 (21.4%), (*p* = 0.3)) with elevated baseline and available follow-up (median 17 weeks (IQR 15–22)) CRP levels in the initial response period. In the maintenance period, CRP values were available for 17 patients with a median follow up of 55 weeks (IQR 49.5–55), and they normalized in 9/17 (52.9%) of those with an elevated CRP at baseline (6/11 (54.5%) and 3/6 (50%), (*p* = 0.9) (Figure 3).

FCP levels were available in 38 out of the 59 patients and were elevated in 26 (68.4%) at treatment onset (14/20 (70%) and 15/22 (66.6%), on anti-TNF and UST therapy, respectively (*p* = 0.9)). FCP normalized in 4/18 (22.2%) patients (2/10 (20%) and 2/8 (25%), (*p* = 0.8)) with elevated baseline and available follow-up (median 16 weeks (IQR 14–20.5)) FCP levels in the initial response period. FCP values at the maintenance period were available for 10 patients, and they normalized with a median follow-up of 52 weeks (IQR 49–55) in 3/10 (30%) of those with an elevated FCP at baseline (1/6 (16.6%) and 2/4 (50%), (*p* = 0.4) (Figure 3).

### 3.3. Discontinuation of Therapy

**Figure 4:**
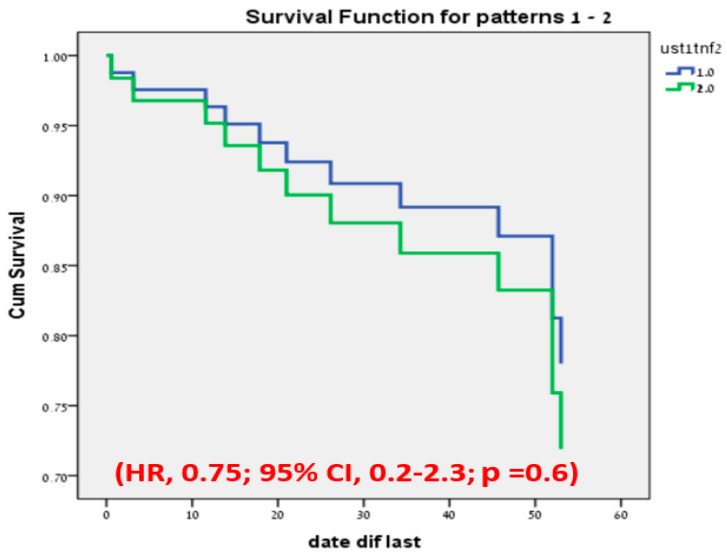
Cox regression test of treatment discontinuation-free analysis; UST1, ustekinumab; TNF2, anti-tumor necrosis factor; HR, hazard ratio; CI, confidence interval; *p*, *p*-value.

The overall treatment discontinuation was observed in 12 patients, with a median follow-up duration of 23.5 (IQR 6.5–50.5) weeks. A total of 7 patients out of 31 (22.5%) on anti-TNF with a median time of 21 weeks (IQR 14–46) and 4/28 (14.2%) on UST therapy with a median time of 34 weeks (6.5–52.5) discontinued treatment without any significant difference between the groups (*p* = 0.6) (Figure 4).

The main reason for the discontinuation of both treatment groups was a lack of response (anti-TNF: 5/7 (71.4%), UST:3/4 (75%). The rest discontinued the treatment because of: patient decision (n = 1), surgery (n = 1), and immunogenicity (n = 1). During the follow-up period, 28% of the patients required dose escalation (32% vs. 21.4% on anti-TNF and UST therapy, respectively; (*p* = 0.4)) without any significant change in the discontinuation rate in the booth subgroups (30% vs. 16.6%, respectively; *p* = 0.6).

### 3.4. Safety

The adverse events that were documented during the entire follow-up period are listed in Table 2. Overall, 7 (10%) patients reported adverse events (4 (10.8%) patients on anti-TNF and 3 (9%) on UST). Adverse events were a cofactor for therapy discontinuation in 4 patients as is described in Table 2. Five patients out of the 70 (7.1%) required surgical intervention (2 (5.8%) and 3 (10.3%) on anti-TNF and UST therapy, respectively, (*p* = 0.5)). Ten patients (14.2%) were hospitalized at least once (5/37 (14.7%) and 5/33 (17.2%), respectively, (*p* = 0.8).

## 4. Discussion

Our study is a comparative real-world study in CD that attempts to address an important evidence gap: to compare the effectiveness and safety of anti-TNF agents and UST as a second-line biologic after VDZ failure. Our retrospective data from 59 CD patients demonstrated that second-line anti-TNF-treated patients had similar rates of clinical effectiveness and adverse event to second-line UST cohort in both induction and maintenance period (52 weeks’ post-treatment initiation). These results support the effectiveness and safety of anti-TNF and UST as a second-line biologic treatment after VDZ failure.

VDZ is efficacious in both TNF-naïve and TNF-failure populations [5,7,8,9,10]. Despite the adequate efficacy about 39–42% of the patients will stop the therapy mainly due to loss of response and adverse events [28,29,30]. The choice of drug positioning is based upon clinicians’ experience, costs and drug availability in the region. Moreover, data regarding efficacy of anti-TNF and UST therapy after VDZ failure as a first-line therapy are very limited.

There is only one study that evaluated the efficacy and safety of anti-TNF as a second-line biologic after VDZ failure in pediatric IBD patients. In particular, Dolinger, M. et al. [31] included 21 pediatric patients with mostly colonic disease (19/21). The study included only six CD patients with numerically lower remission rates than in UC/IBD-U (50% vs. 80% *p* = 0.27). Three out of the six patients discontinued anti-TNF therapy after a median duration of 15 (7–24) weeks. No serious adverse events, hospitalizations or serious infections attributable to anti-TNF therapy were described in the study. In our study, the overall clinical remission was achieved in 49.1% after the induction period without significantly difference between anti-TNF and UST. The remission rate remained high in the maintenance period (60.6%) and steroid-free clinical remission was achieved in a greater proportion of patients (52.1%).

Earlier studies have shown the effectiveness of UST in CD patients refractory to anti-TNFs. However, these studies do not describe the effectiveness of UST as second-line biologic after VDZ failure as a first-line therapy [32,33,34,35].

In our study 20.3% (12/59) of the patients discontinued the therapy, while the majority continued the therapy with an acceptable response (78.7%%) and remission rates (60.6%). Effectiveness of anti-TNF and UST as a second-line biologic after VDZ failure suggest that the effectiveness may not be compromised by prior VDZ exposure, as shown in previous studies [36,37,38].

Only four adverse events were as cofactors for therapy discontinuation (one anti-TNF, three UST), adding further to the literature supporting anti-TNF and UST safety profiles [39,40].

Important limitations of the study need to be acknowledged. The major limitation of this study is the retrospective multicenter nature real world (RWE) evidence of the data collection, with the potential biases including recall bias; missing some laboratory data, including drug levels and anti-drug antibodies; heterogeneity in the scheduled visit dates and reporting bias of side effects which patients may not have reported or may not have been recorded. These limitations are not unique to our study and are similar to most multicenter RWE series. The second limitation is the lack of data pertaining to the response of extra intestinal manifestations and perianal disease. Further limitations include the relatively modest number of patients, that may be secondary to the limited indication of VDZ as a first-line therapy in many countries. Due to this low number of patients, we were unable to calculate the predictors of clinical response. The study cohort was narrow in terms of age (51.8 years old (IQR 32–70)) and may not well reflect the responses of all patients. There is no representation for the young under the age of 30. The time definition was also challenging to implement due to different follow-up and treatment approaches in the different centers. Thirty-seven percent of the cohort had available endoscopic follow-up examinations, with a time lag between the endoscopies and a median of 44 weeks (IQR 21–57.5).

Despite the aforementioned limitations, our study is the largest to data addressing the effectiveness of second-line biologic after VDZ failure. Further research would be required to identify the most effective treatment regimen for Crohn’s disease patients failing VDZ as first-line biological therapy.

In conclusion, second line therapy after VDZ failure therapy was effective in more than 60% of the patients with CD. No differences in effectiveness were detected between the use of anti-TNF and UST as a second line.

## Figures and Tables

**Table 1:** Patients’ demographic and clinical characteristics.

	Overall	UST	Anti-TNF	*p*-Value
Patient number, N (%)	59	28 (47.5)	31 (52.5)	0.7
Demographics				
Mean (SD) age, years	52 (19)	58 (17.5)	46 (19)	0.9
Gender [male], n(%)	25 (42.4)	12 (42.8)	13 (41.9)	0.9
Gender [female], n(%)	34 (57.6)	16 (57.1)	18 (58)	0.9
Smoking status				
Current, n (%)	9 (15.2)	5 (17.8)	4 (12.9)	0.6
Former, n (%)	13 (22)	8 (28.5)	5 (16.1)	0.3
Never smoked, n (%)	37 (62.8)	15 (53.5)	22 (70.9)	0.5
Clinical characteristics				
Disease duration, years (IQR)	35 (23–56.5)	43 (25–60)	36 (21–48)	0.4
Disease location				
Ileal	19 (32.4)	11 (39.2)	8 (25.8)	0.4
Colonic	18 (30.5)	9 (32.1)	9 (29)	0.8
Ileocolonic	21 (35.5)	7 (25)	14 (45.1)	0.2
Upper-GI	1 (1.6)	1 (3.5)	0 (0)	0.4
Behavior				
Non-stricturing, non-penetrating	39 (66.2)	20 (71.4)	19 (61.2)	0.7
Stricturing	16 (27.1)	7 (25)	9 (29)	0.7
Penetrating	4 (6.7)	1 (3.5)	3 (9.6)	0.3
Perianal disease	5 (8.4)	3 (10.7)	2 (6.4)	0.5
History of surgery	17 (28.8)	8 (28.5)	9 (29)	0.9
Mean vedolizumab therapy duration, months (IQR)	12 (5–17)	14.3 (6–18.7)	10 (4–14)	0.1
Disease activity at treatment onset				
HBI median, (IQR)	8 (6–9)	9 (7–10.7)	7 (6–9)	0.6
Elevated CRP, n (%)	36 (66.6)	16 (59.2)	20 (74)	0.7
Elevated FCP, n (%)	26 (68.4)	12 (66.5)	14 (70)	0.9
Concomitant corticosteroid	23 (38.9)	13 (46.4)	10 (32.2)	0.6
Concomitant immunomodulators	6 (10.1)	1 (3.5)	5 (16.1)	0.1

Abbreviations: n, number; UST, ustekinumab; anti-TNF, anti-tumor necrosis factor; IQR, interquartile range; Upper-GI, upper gastrointestinal; HBI, Harvey–Bradshaw Index, CRP, C-reactive protein; FCP, Fecal Calprotectin; SD, Standard deviation.

**Table 2:** Adverse events.

Patients Number	Drug	Adverse Events	Stopped Therapy	Required Hospitalization
Patient 1	UST	Rash in the extremities and itchy	Yes	No
Patient 2	Anti-TNF	Allergic reaction to anti TNF	No	No
Patient 3	Anti-TNF	Fatigue, arthralgia, myalgia	No	No
Patient 4	Anti-TNF	Worsening in ITP	Yes	Yes
Patient 5	UST	Uneasiness, loss of strength	Yes	No
Patient 6	Anti-TNF	Pancreatitis	No	Yes
Patient 7	UST	Lung cancer	Yes	Yes

Abbreviations: UST, ustekinumab; anti-TNF, anti-tumor necrosis factor; ITP, Immune Thrombocytopenia.

## Data Availability

No new data were created or analyzed in this study. Data sharing is not applicable to this article.
